# Nasopharyngeal microbiota in infants and changes during viral upper respiratory tract infection and acute otitis media

**DOI:** 10.1371/journal.pone.0180630

**Published:** 2017-07-14

**Authors:** Tasnee Chonmaitree, Kristofer Jennings, Georgiy Golovko, Kamil Khanipov, Maria Pimenova, Janak A. Patel, David P. McCormick, Michael J. Loeffelholz, Yuriy Fofanov

**Affiliations:** 1 Department of Pediatrics, University of Texas Medical Branch, Galveston, TX, United States of America; 2 Department of Pathology, University of Texas Medical Branch, Galveston, TX, United States of America; 3 Department of Preventive Medicine and Community Health, University of Texas Medical Branch, Galveston, TX, United States of America; 4 Sealy Center for Structural Biology, University of Texas Medical Branch, Galveston, TX, United States of America; Instituto Butantan, BRAZIL

## Abstract

**Background:**

Interferences between pathogenic bacteria and specific commensals are known. We determined the interactions between nasopharyngeal microbial pathogens and commensals during viral upper respiratory tract infection (URI) and acute otitis media (AOM) in infants.

**Methods:**

We analyzed 971 specimens collected monthly and during URI and AOM episodes from 139 infants. The 16S rRNA V4 gene regions were sequenced on the Illumina MiSeq platform.

**Results:**

Among the high abundant genus-level nasopharyngeal microbiota were *Moraxella*, *Haemophilus*, and *Streptococcus* (3 otopathogen genera), *Corynebacterium*, *Dolosigranulum*, *Staphylococcus*, *Acinetobacter*, *Pseudomonas*, and *Bifidobacterium*. Bacterial diversity was lower in culture-positive samples for *Streptococcus pneumoniae*, and *Haemophilus influenzae*, compared to cultured-negative samples. URI frequencies were positively associated with increasing trend in otopathogen colonization. AOM frequencies were associated with decreasing trend in *Micrococcus* colonization. During URI and AOM, there were increases in abundance of otopathogen genera and decreases in *Pseudomonas*, *Myroides*, *Yersinia*, *and Sphingomonas*. Otopathogen abundance was increased during symptomatic viral infection, but not during asymptomatic infection. The risk for AOM complicating URI was reduced by increased abundance of *Staphylococcus and Sphingobium*.

**Conclusion:**

Otopathogen genera played the key roles in URI and AOM occurrences. *Staphylococcus* counteracts otopathogens thus *Staphylococcal* colonization may be beneficial, rather than harmful. While *Sphingobium* may play a role in preventing AOM complicating URI, the commonly used probiotic *Bifidobacterium* did not play a significant role during URI or AOM. The role of less common commensals in counteracting the deleterious effects of otopathogens requires further studies.

## Introduction

Otitis media is a common childhood disease; it is the leading cause of doctors’ visits by children, and the most frequent reason children consume antibiotics or undergo surgery [1–3]. Acute otitis media (AOM) occurs as a complication of viral upper respiratory tract infection (URI) [4]. AOM is a polymicrobial disease; its pathogenesis involves complex interactions between bacteria, viruses, and the host inflammatory response [5, 6].

During the first months of life the young infant’s upper respiratory tract gradually acquires complex microbial communities, including pathogens and commensals [7–9]. It is well known that the three otopathogens (*Streptococcus pneumoniae*, non-typeable *Haemophilus influenzae*, and *Moraxella catarrhalis*) colonize the nasopharynx from early infancy but bacterial AOM only occurs after viral URI [10–12]. Less is known about the colonization of commensals and their dynamics during viral URI. Existing data suggest interference between otopathogens and commensals [13–15]. Therefore, enhancement of commensal colonization may interfere with otopathogen colonization leading to disease prevention. Investigators have studied the effect of selected commensal bacteria, e.g. *Bifidobacterium* and *Lactobacillus*, the so-called ‘probiotic bacteria’, in preventing URI and recurrent AOM, but the results have been mixed [16–18]. It is possible that effective probiotic component may require combinations of protective commensals. Refinement of probiotics for prevention of URI and AOM depends on better knowledge and understanding of respiratory tract microbiota and pathogen, commensal, and viral interactions.

There have been recent studies of respiratory microbiota in children during URI and AOM [19–21]. These studies were cross-sectional, with no specific viral data, and the children were mostly older than 6 months. Others have performed longitudinal studies of respiratory microbiota but did not focus on viral URI and AOM [7–9, 22]. The purpose of our study was to characterize nasopharyngeal microbiota in infants followed from near birth to the first AOM episode or 12 months of age and elucidate how changing patterns of nasopharyngeal bacterial colonization lead to susceptibility to viral URI and AOM development.

## Methods

### Study design, subjects and specimens

The subjects were part of a prospective, longitudinal study (2008–2014) of infants in the first year of life to evaluate the prevalence and risks for URI and AOM development [11, 23]. Nasopharyngeal (NP) specimens analyzed in this study included available specimens (average 7 / per subject) from an approximately equal number of subjects with and without AOM. This study was approved by the University of Texas Medical Branch (UTMB) Institutional Review Board and conformed to the human experimentation guidelines of the United States Department of Health and Human Services. Written informed consent was obtained from the parents/guardians of all subjects.

In brief, healthy infants were enrolled from near birth (< 1 month) and completed the study after the first AOM episode was diagnosed, or at age 12 months without AOM; all subjects were followed at least 6 months. Specimens were collected monthly during months 1–6, month 9, and during URI and AOM. Details on data collection, URI and AOM diagnostic criteria, follow-up, and specimen processing are in **S1 File**.

### DNA extraction/ amplification and sequencing

From the nasopharyngeal samples, DNA was extracted using the PowerMag PowerMicrobiome DNA/RNA Isolation kit (MoBio) in the STARlet platform (Hamilton Robotics). The conserved hypervariable 16S rDNA V4 region was amplified by PCR. The amplicons were sequenced using 2 x 250 bp paired end protocol on the MiSeq platform (Illumina), yielding pair-end reads that overlap by ~247 bps. Following sequencing, raw BCL files were retrieved from the MiSeq platform and called into FASTQs by Casava v1.8.3 (Illumina). The read pairs were demultiplexed based on unique molecular barcodes allowing for up to 1 substitution mismatch and reconstituted into two FASTQ files for each. The resulting 250 base long paired end reads were merged together based on the overlapping region. These laboratory procedures were performed at the Alkek Center for Metagenomics and Microbiome Research, Baylor College of Medicine (Joseph Petrosino, Director)[24].

### Sequence analysis

To identify the presence of known bacteria and archaea, subsequences were analyzed using CLC Genomics Workbench 8.0.1 Microbial Genomics Module (http://www.clcbio.com). Read containing nucleotides below the quality threshold of 0.05 (using the modified Richard Mott algorithm) and reads with two or more unknown nucleotides or sequencing adapters were filtered. All reads were trimmed to 240 bases for operational taxonomic unit (OTU) classification. Reference based OTU picking was performed using the SILVA SSU v119 97% database [25]. Sequences present in more than one copy but not clustered to the database were then placed into de novo OTUs (97% similarity) and aligned against the reference database with 80% similarity threshold. Chimeras were removed from the results if their absolute crossover cost was 3 using a k-mer size of 6.

### Statistics

All analyses were done at the genus level (**S2 File**). The Shannon diversity index was calculated using the entropy function in the entropy library in R statistical package (cran.r-project.org). To account for the within-subject variability component, a mixed model with a random intercept for subject was used. Finally, abundance was calculated using the mean relative abundance across samples. Significance was declared with P < 0.05. Adjustments for multiple testing were done using the Benjamini-Hochberg adjustment for controlling the false discovery rate (FDR); unadjusted results are also shown to highlight how the similar genera had significant differences across multiple modeling structures. All calculations were done in R (version 3.2.2) and associated libraries (lme4, lmerTest, entropy, p.adjust). Detailed statistical methods are provided in **S1 File**.

## Results

### I. Subject characteristics and number of specimens

Characteristics of the subjects and number of specimens are shown in **Table 1**(Metadata are shown in **S3 File**). The first specimen in this study was collected in August 2009, and the last, January 2014. Of 139 subjects, 96% had at least 2 healthy (asymptomatic) samples, 77% had (268) URI/ AOM samples, and 60% had at least 1 healthy sample before URI/ AOM samples.

**Table 1:** Characteristics of subjects and number of specimens.

	Total	Subjects with AOM	Subjects without AOM
Number of subjects	139 (100)	65 (47)^a^	74 (53)
Male	83 (60)^b^	36 (55)	47 (64)
Female	56 (41)	29 (45)	27 (36)
Race			
- White	119 (86)	56 (86)	63 (85)
- African American	18 (13)	9 (14)	9 (12)
- Asian	2 (1)	0	2 (3)
Ethnicity			
- Hispanic/ Latino	77 (56)	35 (54)	42 (57)
- NonHispanic/ Latino	62 (44)	30 (46)	32 (43)
Breastfeeding			
- Exclusive breastfeeding for 6 months	13 (9)^b^	5 (8)	8 (11)
- Exclusive breastfeeding for 3 months	7 (5)	5 (8)	2 (3)
- Exclusive formula feeding	62 (45)	32 (49)	30 (41)
- Mixed feeding	57 (41)	23 (35)	34 (46)
One or more siblings at home (% yes)	55	57	54
Daycare attendance (% yes)	33	22	43
Cigarette smoke exposure (% yes)	23	18	27
Number of Specimens	971^c^	432	539
Average number of specimens per subject	7	6.6	7.3
Age at samples collection^d^			
- 1 month	131	57	74
- 2 months	136	64	72
- 3 months	148	71	77
- 4 months	139	65	74
- 5 months	145	73	72
- 6 months	137	63	74
- 7–12 months	135	39	96
Specimens collected during URI^e^	223	119	104
Specimens collected during AOM	45	45	0
Specimens collected after antibiotic use			
- 7 days	37	30	7
- 14 days	43	36	7
- 1 month	71	57	14
- 2 months	98	72	26

a- row %

b-column % (% within the same category)

c- all specimens sequenced; 23 (2%) of these had <1000 sequence reads were excluded

d- nearest month, both monthly and URI samples

e- median day of URI at the time of sample collection = day 4, excluded samples collected during AOM diagnosis

### II. Nasopharyngeal microbial communities

We obtained a total of 20,976,078 high-quality bacterial sequences comprising 13,982 unique operational taxonomical units (OTUs). Of 971 sequenced samples, 948 (98%) yielded ≥ 1000 sequences (mean = 22,118, median = 19,398, range 1,013–337,402 reads/ sample). Further analyses included only 948 samples with > 1000 sequence reads. Overall, 4 phyla predominated the sequence reads: Proteobacteria (39%), Actinobacteria (26%), Firmicutes (26%), and Bacteroidetes (6%).

#### Diversity of the microbial communities

**Fig 1:**
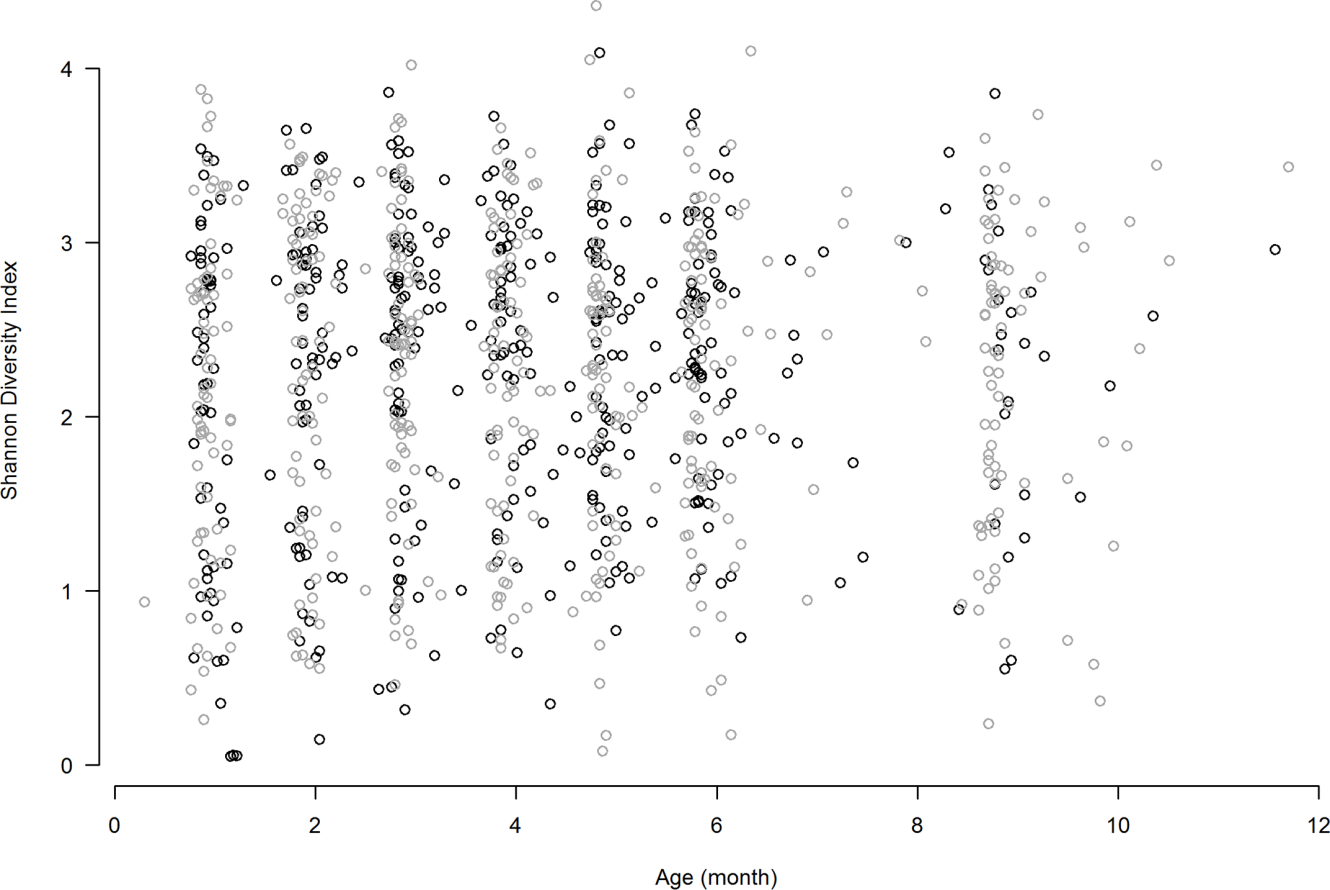
Shannon diversity index by type of samples. Healthy (grey circles) or sick visit (URI only or URI with AOM) samples (black circles).

Alpha diversity, as measure by Shannon Diversity Index (SDI) by age and type of samples (healthy vs URI/ AOM samples) is shown in **Fig 1**. There was no association between with age or sample type (p > 0.2 and p > 0.3, respectively).

**Fig 2:**
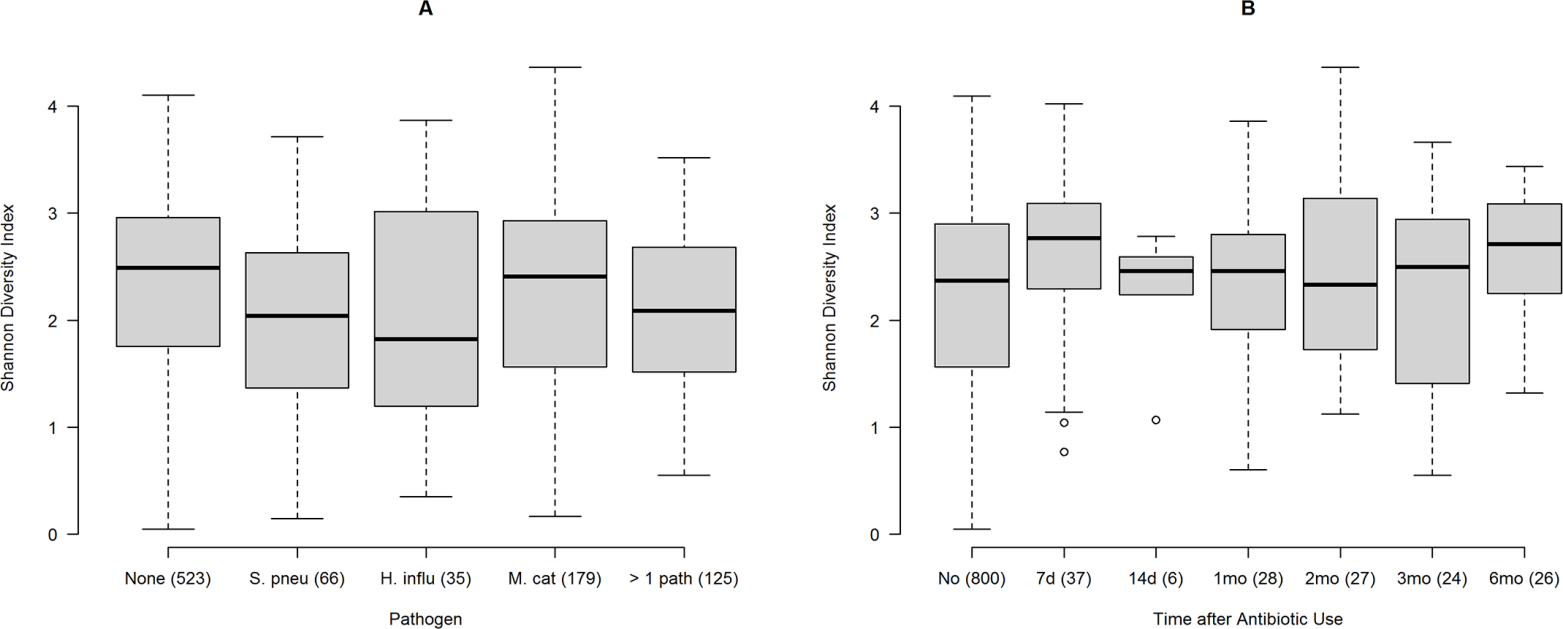
**A-B. Shannon diversity index.** Fig 2A. Shannon Diversity Index by positive culture for otopathogens. Number in parentheses are number of samples with positive cultures. None = negative culture; S. pneu = *Streptococcus pneumoniae*; H. influ = *Haemophilus influenzae*; M. cat = *Moraxella catarrhalis*; > 1 path = positive culture for more than one of these pathogens. Fig 2B. Shannon Diversity Index by specific time point after antibiotic use. Samples were grouped based on the time of antibiotic use before sample collection: 7 days, 14 days, 1 month, 2 months, 3 months, and 6 months, comparison was made with samples collected after no history of antibiotic use.

SDI by bacterial culture results are displayed in **Fig 2A**. The median SDI was 2.49 for samples with no otopathogen, compared to 2.04 in samples positive for *S*. *pneumoniae* only (P = 0.022); 1.82 for *H*. *influenzae* (P = 0.040); 2.41 for *M*. *catarrhalis* (P = 0.620) and 2.09 for 2 or more otopathogens (P = 0.048).

Antibiotic use led to higher diversity overall (p = 0.0029) (**Fig 2B**); diversity was highest at 7d after antibiotic use (P = 0.011). There was no difference in diversity at 14 days, and at 1, 2, or 3 months, but diversity at 6 months of antibiotic use was higher compared to no antibiotic use (P = 0.016). Diversity was not associated with gender (P = 0.22), race (P = 0.88), breastfeeding (P>0.4), or mode of delivery (P = 0.4).

In the samples from the same subject, diversity at baseline (month 1) was positively associated with diversity at later age (P = 0.02; **S1A Fig**). Higher diversity at month 1 was associated with increasing frequencies of URI within the first 6 months (P = 0.036; **S1B Fig**), but baseline diversity was not associated with AOM frequency (P = 0.55; **S1C Fig**).

#### Nasopharyngeal microbiota overall

The top 21 most abundant genus-level microbiota (each accounted for ≥ 0.5% abundance) in 948 samples are shown in **Table 2.** These genera accounted for 70.3% of all identified bacterial genera. Otopathogen genera (*Moraxella*, *Haemophilus*, *and Streptococcus*), *Staphylococcus* and *Pseudomonas* were among the most common pathogen genera, while *Corynebacterium*, *Dolosigranulum*, and *Acinetobacter* accounted for the top 3 commensal genera. Samples from infants with AOM in the first year had significantly higher average abundance of *Haemophilus*, *Enterobacter*, and *Yersinia*, and lower abundance of *Corynebacterium*, and *Pseudomonas* compared to samples from infants without AOM.

**Table 2:** Relative abundance of microbiota in samples from subjects without and with AOM in the first year.

					Samples from	Samples from	Samples from	
Phylum	Class	Order	Family	Genus	all subjects ^a^	subjects without AOM	subjects with AOM	P-value (Age-adjusted)
					(N = 948)	(N = 516)	(N = 432)	
Actinobacteria	Actinobacteria	Corynebacteriales	Corynebacteriaceae	Corynebacterium	17.8%	19.7%	15.5%	0.0071*
Proteobacteria	Gammaproteobacteria	Pseudomonadales	Moraxellaceae	Moraxella	9.7%	9.1%	10.4%	0.4309
Firmicutes	Bacilli	Lactobacillales	Carnobacteriaceae	Dolosigranulum	6.8%	7.8%	5.7%	0.0811
Firmicutes	Bacilli	Bacillales	Staphylococcaceae	Staphylococcus	5.7%	6.0%	5.4%	0.8723
Proteobacteria	Gammaproteobacteria	Pseudomonadales	Moraxellaceae	Acinetobacter	4.1%	4.6%	3.6%	0.2243
Proteobacteria	Gammaproteobacteria	Pasteurellales	Pasteurellaceae	Haemophilus	3.7%	2.6%	5.0%	0.0072*
Proteobacteria	Gammaproteobacteria	Pseudomonadales	Pseudomonadaceae	Pseudomonas	3.5%	4.0%	2.9%	0.0103*
Firmicutes	Bacilli	Lactobacillales	Streptococcaceae	Streptococcus	3.5%	3.3%	3.7%	0.2990
Actinobacteria	Actinobacteria	Bifidobacteriales	Bifidobacteriaceae	Bifidobacterium	2.9%	2.1%	3.9%	**0.0316**
Proteobacteria	Gammaproteobacteria	Enterobacteriales	Enterobacteriaceae	Enterobacter	2.5%	1.9%	3.1%	0.0019*
Actinobacteria	Actinobacteria	Micrococcales	Micrococcaceae	Micrococcus	1.6%	1.6%	1.7%	0.5602
Proteobacteria	Gammaproteobacteria	Chromatiales	Ectothiorhodospiraceae	Arhodomonas	1.5%	1.9%	1.1%	0.6486
Bacteroidetes	Bacteroidia	Bacteroidales	Bacteroidaceae	Bacteroides	1.3%	1.0%	1.6%	0.1554
Firmicutes	Clostridia	Clostridiales	Ruminococcaceae	Incertae Sedis	1.0%	0.7%	1.3%	0.4470
Proteobacteria	Betaproteobacteria	Burkholderiales	Burkholderiaceae	Ralstonia	0.9%	0.9%	0.9%	0.2199
Bacteroidetes	Flavobacteriia	Flavobacteriales	Flavobacteriaceae	Myroides	0.8%	1.1%	0.5%	**0.0202**
Proteobacteria	Gammaproteobacteria	Enterobacteriales	Enterobacteriaceae	Pantoea	0.6%	0.5%	0.8%	0.8054
Proteobacteria	Gammaproteobacteria	Enterobacteriales	Enterobacteriaceae	Yersinia	0.6%	0.6%	0.7%	0.0051*
Firmicutes	Clostridia	Clostridiales	Clostridiaceae 1	Clostridium sensu stricto 1	0.6%	0.6%	0.6%	0.0688
Proteobacteria	Alphaproteobacteria	Sphingomonadales	Sphingomonadaceae	Sphingomonas	0.6%	0.6%	0.6%	0.6080
Proteobacteria	Alphaproteobacteria	Sphingomonadales	Sphingomonadaceae	Sphingobium	0.6%	0.6%	0.6%	0.1464

a-66 subjects had AOM in the first year; 73 subjects did not

Data presented as an average value of relative abundance of the specific genus within each sample

All results which are still significant after adjustment for multiple testing indicated by *.

#### Effect of antibiotic on the nasopharyngeal microbiota

Of 948 samples, 148 (16%) were collected within 7 days to 6 months of antibiotic use. The usual antibiotic course was 7–10 days; the most commonly used antibiotic was amoxicillin. The abundance of microbiota in relation to the time to prior antibiotic use is shown in **S2 Fig**. Antibiotics did not significantly affect the otopathogen genera but significantly decreased: 1) *Corynebacterium* (P = 0.0165) and *Dolosigranulum* (P = 0.0084) within 7 days; 2) Enterobacter between 7 and 14 days (P = 0.0347); and 3) *Staphylococcus* at between 14 days and 1 month (P = 0.0161). Antibiotic increased the abundance of *Bifidobacterium* (P = 0.0146) and Firmicutis *Incertae Sedis* (P = 0.0140) at the 7day time point.

### III Effect of URI/ viral infection on microbiome composition

#### Microbiota in healthy vs sick visit (URI with and without AOM) samples

**Table 3**compares microbiome composition in healthy vs sick visit samples. The abundance of all 3 otopathogen genera was significantly higher in sick visit samples, while there was a significant reduction in *Pseudomonas*, *Myroides*, *Yersinia*, and *Sphingomonas*. Significance remained after adjustment for multiple comparisons for higher abundance of *Moraxella* and lower abundance of *Yersinia* in sick visit samples. Comparison between microbiota in specific sample types (healthy vs URI, healthy vs AOM, and URI vs AOM) are shown in **Tables A-C in S4 File**, respectively. Comparing AOM samples to healthy samples, there was higher abundance of *Moraxella* and *Haemophilus*.

**Table 3:** Relative abundance of microbiota in healthy samples compared to sick visit (URI with or without AOM) samples.

Genus	Total	Healthy samples	URI / AOM samples	P-value
	(N = 948)	(N = 685)	(N = 263)	(age-adjusted)
Corynebacterium	17.8%	18.3%	16.4%	0.6094
Moraxella	9.7%	8.4%	13.1%	0.0006*
Dolosigranulum	6.8%	6.8%	7.0%	0.4009
Staphylococcus	5.7%	6.2%	4.5%	0.0755
Acinetobacter	4.1%	4.3%	3.7%	0.0534
Haemophilus	3.7%	2.7%	6.3%	**0.0314**
Pseudomonas	3.5%	3.7%	2.9%	**0.0149**
Streptococcus	3.5%	3.1%	4.3%	**0.0278**
Bifidobacterium	2.9%	2.8%	3.4%	0.2694
Enterobacter	2.5%	2.5%	2.4%	0.4921
Micrococcus	1.6%	1.6%	1.8%	0.1119
Arhodomonas	1.5%	1.6%	1.3%	0.6138
Bacteroides	1.3%	1.4%	0.9%	0.1618
Incertae Sedis	1.0%	1.0%	1.0%	0.0736
Ralstonia	0.9%	1.0%	0.8%	0.5836
Myroides	0.8%	0.9%	0.6%	**0.0150**
Pantoea	0.6%	0.7%	0.4%	0.3637
Yersinia	0.6%	0.7%	0.5%	0.0010*
Clostridium sensu stricto 1	0.6%	0.6%	0.4%	0.3139
Sphingomonas	0.6%	0.6%	0.5%	**0.0448**
Sphingobium	0.6%	0.6%	0.6%	0.2267

* Result still significant at the 0.05 level after adjustment for multiple testing

#### Effect of virus infections

Virus data were available in 890 of 948 samples (94%); one or more viruses were detected from 42% of samples (500 viruses). Rhinovirus was the most common virus (39%); human coronavirus was detected in 20%; enterovirus, 11%; parainfluenza, 9%; adenovirus, 6%; respiratory syncytial virus, 6%; bocavirus, 4%; metapneumovirus, 4%; and influenza virus, 1%. **Table 4**compares microbiota in virus-positive vs virus-negative samples. To evaluate the effect of symptomatic virus infection, we compared microbiota in virus-positive URI samples with and virus-positive healthy samples (**Table A in S5 File**), and with virus-negative healthy samples (**Table B in S5 File**). Symptomatic virus infection samples had significant increased abundance of *Moraxella* and *Streptococcus*. Effect of asymptomatic viral infection is shown in **Table C in S5 File**. Rhinovirus was the most common virus detected (n = 193) regardless of symptoms; **Table D in S5 File** compares microbiota in rhinovirus-positive and rhinovirus-negative samples.

**Table 4:** Relative abundance of microbiota in virus-negative and virus-positive samples.

Genus	All samples	Virus-negative samples	Virus-positive samples	P-value (age-adjusted)*
	(N = 872)	(N = 509)	(N = 363)	
Corynebacterium	17.8%	18.8%	16.4%	0.2444
Moraxella	10.0%	8.5%	12.0%	**0.0143**
Dolosigranulum	6.9%	7.4%	6.3%	0.2987
Staphylococcus	5.8%	6.3%	5.2%	0.9983
Acinetobacter	4.1%	4.3%	3.9%	0.5937
Haemophilus	3.8%	2.5%	5.7%	0.2414
Streptococcus	3.5%	2.7%	4.6%	0.9299
Pseudomonas	3.5%	3.6%	3.4%	**0.0027**
Bifidobacterium	3.0%	2.8%	3.3%	0.6708
Enterobacter	2.5%	2.6%	2.5%	0.3380
Micrococcus	1.6%	1.7%	1.5%	0.7034
Bacteroides	1.3%	1.5%	1.1%	0.5020
Arhodomonas	1.2%	1.5%	0.9%	0.2255
Incertae Sedis	1.0%	0.9%	1.2%	0.9476
Ralstonia	0.9%	1.0%	0.8%	0.3378
Myroides	0.9%	1.1%	0.7%	**0.0435**
Pantoea	0.7%	0.7%	0.6%	0.1105
Yersinia	0.6%	0.6%	0.6%	0.6689
Clostridium sensu stricto 1	0.6%	0.7%	0.5%	0.9707
Sphingobium	0.6%	0.5%	0.6%	0.9869
Sphingomonas	0.6%	0.6%	0.5%	0.1843

* All P-values were > 0.05 after adjustment for multiple testing

### IV Microbiome composition and frequencies of URI and AOM

We determine the relationship between nasopharyngeal microbiota and URI/ AOM frequencies by comparing the linear trend (from 1–6 months) in microbial presence in samples collected from infants with frequent URIs early in life (e.g. 1, 2, 3, and 4 URI episodes) vs those without URI (control group) (**S1 Table**). In the first 3 months, 168 infants had ≥ 1 URI; in the first 6 months, 105 infants had 1, 76 had two, 41 had three, and 39 had ≥ 4 URI episodes. Infants with more URIs in the first 3 months had increasing abundance of *Streptococcus* (Linear Trend P = 0.0112,), while infants with more URI in the first 6 months had increasing abundance of *Moraxella* and *Haemophilus*, compared to controls. There was no significant difference in abundance of *Staphylococcus* or other commensals between groups. A total of 68 infants had ≥ 1 AOM in the first 6 months. Increased number of AOM episodes in the first 6 months was associated with decreasing *Micrococcus* abundance; there was no significant trend seen with other genera.

### V. Changes in microbiome composition during transition from URI to AOM

The availability of longitudinal samples from the subjects allowed us to determine the changes during transition from URI to AOM. We first studied microbiota in samples collected within the first 7 days of URI onset (N = 184); samples were compared based on the follow-up outcome of URI (resolved vs complicated by AOM). Of these, 167 were URI samples from cases that resolved without AOM complication; 17 were from cases that were later complicated by AOM. **S2 Table** compares microbiota in URI samples from cases that were complicated by AOM vs those that resolved. Although there was higher abundance of *Streptococcus* in the AOM group, the difference did not reach statistical difference when adjusted for age.

**Fig 3:**
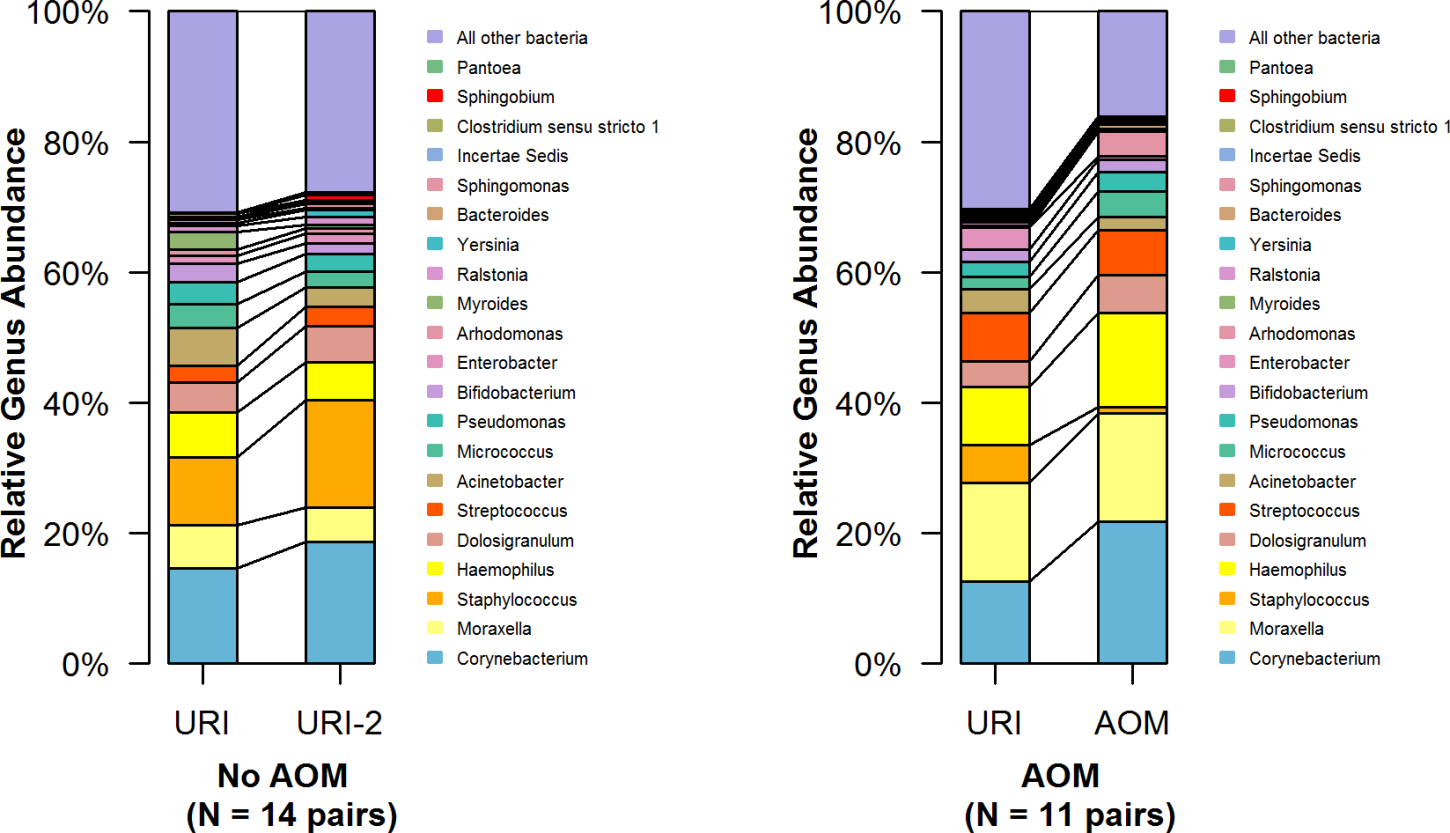
Comparison of the most abundant genera in paired samples collected ≤ 7 days apart. The left set represents data from 14 pairs from subjects with URI that resolved without AOM complication. The right set represents data from 11 pairs from subjects with URI that were complicated with AOM (all within 3 days of the first URI samples).

We then studied paired samples from the same subjects collected ≤ 7 days apart; the first sample of the pair was collected as soon as possible and within 7 days of URI onset. Available data included 2 sets of paired samples; the first set of 14 pairs were URI samples collected 1–7 days apart (mean = 3 days) that did not result in AOM; the average age at the sample collection sets was 3.6 (median = 1.9) months. The second set of 11 paired samples, URI was complicated by AOM within 3 days (follow-up samples = AOM samples); the average age was 5.2 (median = 4.9) months. Significant increases in *Staphylococcus* (P = 0.007) and *Sphingobium* (P = 0.037) were detected in paired samples of URI that recovered, compared to those that resulted in AOM (**Fig 3**and **S3 Table**).

## Discussion

We studied nasopharyngeal microbiota in nearly one thousand samples, collected longitudinally during health and disease (URI and AOM) from one month of age through the first year. Our results suggested that bacterial otopathogen genera (*Haemophilus*, *Streptococcus*, *and Moraxella*) played the key role in the disease process. The increasing trend in colonization of these otopathogen genera was also correlated positively with frequencies of URI, and their presence was associated with URI symptom expression during viral infection. Interestingly, *Staphylococcus*, but not the commonly used probiotic bacterium *Bifidobacterium*, played an important role in counteracting the deleterious effect of otopathogens.

The availability of comprehensive clinical, bacteriologic, and virologic data allowed us to determine the significance of pathogens, commensals, and their interactions with viruses during URI and AOM. Microbiome analyses further expanded the knowledge on commensal bacteria not traditionally cultured or non-culturable. During symptomatic viral infection, there were significant increases in relative abundance of otopathogen genera, while there was no significant change in otopathogen or commensals during asymptomatic viral infection. The finding on symptoms associated with increased otopathogen abundance agrees with a finding from a recent study suggesting that both viruses and bacteria contributed to acute upper and lower respiratory tract symptoms [9]. Microbiome stability found during asymptomatic viral infection helps explain why asymptomatic viral infection did not lead to AOM, which we previously reported from this subject cohort [23].

When comparing microbiota from paired-samples collected during URI episodes that resolved to those complicated by AOM, we found that increased *Staphylococcus* and *Sphingobium* abundance prevented the transition from URI to AOM. It must be pointed out that the average age at the time of sample collection was older in the AOM group compared to no AOM group. It is known that infants <6 months are less often diagnosed with AOM compared to older infants. Our data, although from a small number, suggest that one of the factors preventing AOM development in young infants is colonization with *Staphylococcus*, which helps counteract with the otopathogens. Our previously published report from this cohort [26] has shown the highest *S*. *aureus* colonization rate (25%) in samples collected at age 1 month, with declining in rate to 12% by age 6 months, along with increasing rates of colonization of *S*. *pneumoniae*, *H*. *influenzae*, and *M*. *catarrhalis*. Others have also shown the negative association between *S*. *aureus* colonization and otopathogen colonization [27–29]. Because *S*. *aureus* colonization in young infants is not associated with invasive infections [26–30], *Staphylococcus* colonization may be beneficial than harmful to these young infants. Not only *Staphylococcus* protected them from otopathogen colonization but it also helped prevent the transition from URI to AOM.

In other studies, nasopharyngeal bacterial commensals were studied mainly in children > 6 months of age, and the samples were often collected during disease state [19, 20, 31]. Our data were from infants, 86% of whom were 1–6 months. Therefore, commensal genera in our subjects were somewhat different than those reported from older and/ or sick children. In a recent study [7], samples were collected from 102 healthy individuals at 24–36 hours after delivery, 7 and 14 days, and 1, 2, 3 4 and 6 months; the commensal genera shown were more comparable to ours. Not only age has been shown to affect nasopharyngeal microbiota, numerous other factors such as mode of delivery, infant feeding type, antibiotic use, vaccines, etc. have also been found to affect microbiota in healthy children [7, 20, 32, 33]. Nevertheless, *Corynebacterium* and *Dolosigranulum* have consistently been found to be the two most common commensal genera in the nasopharynx of children.

During active disease processes when pathogens become predominant, the relative abundance of commensals is reduced, but specific commensals are affected differently. We searched for commensal genera that were affected by the surge in otopathogens; we did not find significant effect on high abundant genera such as *Corynebacterium* or *Dolosigranulum* during URI and/or AOM. Aside from *Staphylococcus*, *Proteobacteria* such as *Acinetobacter*, *Pseudomonas*, *Yersinia*, and *Bacteroides (Myroides)* were significantly reduced. Decreasing trend in colonization with *Micrococcus* was associated with increased AOM frequencies in the first 6 months. The role of these less common commensals in counteracting with otopathogens and preventing AOM deserves further investigations.

This study is limited in that we reported our data to the genus-level, which is not uncommon for this type of study. The otopathogen genera reported may have contained more than the specific otopathogen species. For example, the *Streptococcus* genus may also contain alpha *Streptococci* in addition to *S*. *pneumoniae*. However, Bosch et al. [7] have shown that *S*. *pneumoniae* represented a high proportion of the *Streptococcus* genus in the samples collected from infants > 1 month. Similarly, Teo et al. [9] showed in a study of infants 2–12 months of age that otopathogen genera were dominated by *S*. *pneumoniae*, *H*. *influenzae*, *and M*. *catarrhalis*. In addition, our bacterial culture data confirmed the relationship between the increased bacterial otopathogen colonization during URI and laboratory-confirmed viral infections.

We studied nasopharyngeal microbiota in a search for important commensal bacteria that may be used as effective intranasal probiotics to prevent AOM. The ideal probiotics should be bacteria that have the most interference (counteracting) with pathogens and cause no harm. Previous studies have used both oral and intranasal probiotics containing alpha *streptococci*, *Lactobacillus rhamnosus* GG and *Bifidobacterium*; results have been mixed [16, 34]. We have not found the *Lactobacillus* genus to be a major nasopharyngeal commensal and we do not have the data on alpha *streptococci*. *Bifidobacterium* was commonly found in our samples, ranked 5th among the commensal genera; we did not find it play a role during URI or AOM process. Furthermore, *Bifidobacterium* was found more commonly in samples from infants with AOM in the first year of life. Our data suggest that intranasal use of *Bifidobacterium* may not be helpful as a probiotic. *Corynebacterium*, the most common commensal was found to be significantly lower in samples from infants with AOM in the first year (Table 2), but we observed no other significant difference during the URI or AOM process. Other bacteria that played a reverse role with otopathogens such as *Yersinia* and *Pseudomonas* may not be appropriate probiotics for their possible roles as pathogens.

The mechanisms of AOM pathogenesis are complex; numerous host and environmental factors, as well as interactions between viruses, otopathogenic bacteria and commensals play roles in AOM development. The results of this study emphasize the importance of otopathogens and suggest that prevention of nasopharyngeal otopathogen colonization and viral infection will be the key to preventing AOM.

## Supporting information

S1 Fig Association between diversity at baseline (month 1) and later diversity, URI and AOM frequencies. **A.** Association between baseline diversity and diversity at later age (month 6), P = 0.02. **B.** Association between diversity at baseline (month 1) and number of URI in the first 6 months. Higher diversity at month 1 was associated with increasing frequencies of URI within the first 6 months (P = 0.036). **C.** Association between diversity at baseline (month 1) and number of URI in the first 6 months (P = 0.55).
(TIF)

S2 Fig Antibiotic use and changes in relative abundance. The time point indicates days or months of antibiotic use prior to nasopharyngeal sample collection. Numbers in parentheses are number of samples collected within specific time of antibiotic use.
(TIF)

S1 Table Susceptibility to URI and AOM and trends in microbial genera change during in the first 6 months of life. (DOC)

S2 Table Microbiome composition in URI samples that were complicated by AOM vs URI that resolved. (DOC)

S3 Table Changes in microbiome between paired samples: URI—resolved vs URI to AOM. (DOC)

S1 File Supplemental detailed methods. (DOC)

S2 File OTU table. (XLSX)

S3 File Metadata. (XLS)

S4 File Comparison of relative abundance of microbiota in healthy, URI and/ or AOM samples. Table A. Microbiota in healthy samples compared to URI samples. Table B. Microbiota in healthy samples compared to AOM samples. Table C. Microbiota in URI samples compared to AOM samples.
(DOC)

S5 File Comparison of relative abundance of microbiota in virus-negative, virus-positive vs healthy samples with and without URI symptoms. Table A. Microbiota in virus-positive healthy samples and virus-positive URI samples. Table B. Microbiota in virus-negative healthy samples and virus-positive URI samples. Table C. Effect of asymptomatic virus infection: microbiota in healthy virus-negative vs healthy virus-positive samples. Table D. Microbiota in rhinovirus-negative samples and rhinovirus-positive samples.
(DOC)
