# Supplementary figures and images for: Nasopharyngeal microbiota in infants and changes during viral upper respiratory tract infection and acute otitis media
Source: PLoS One. 2017 Jul 14;12(7):e0180630. doi: 10.1371/journal.pone.0180630 (PMC5510840; doi:10.1371/journal.pone.0180630)

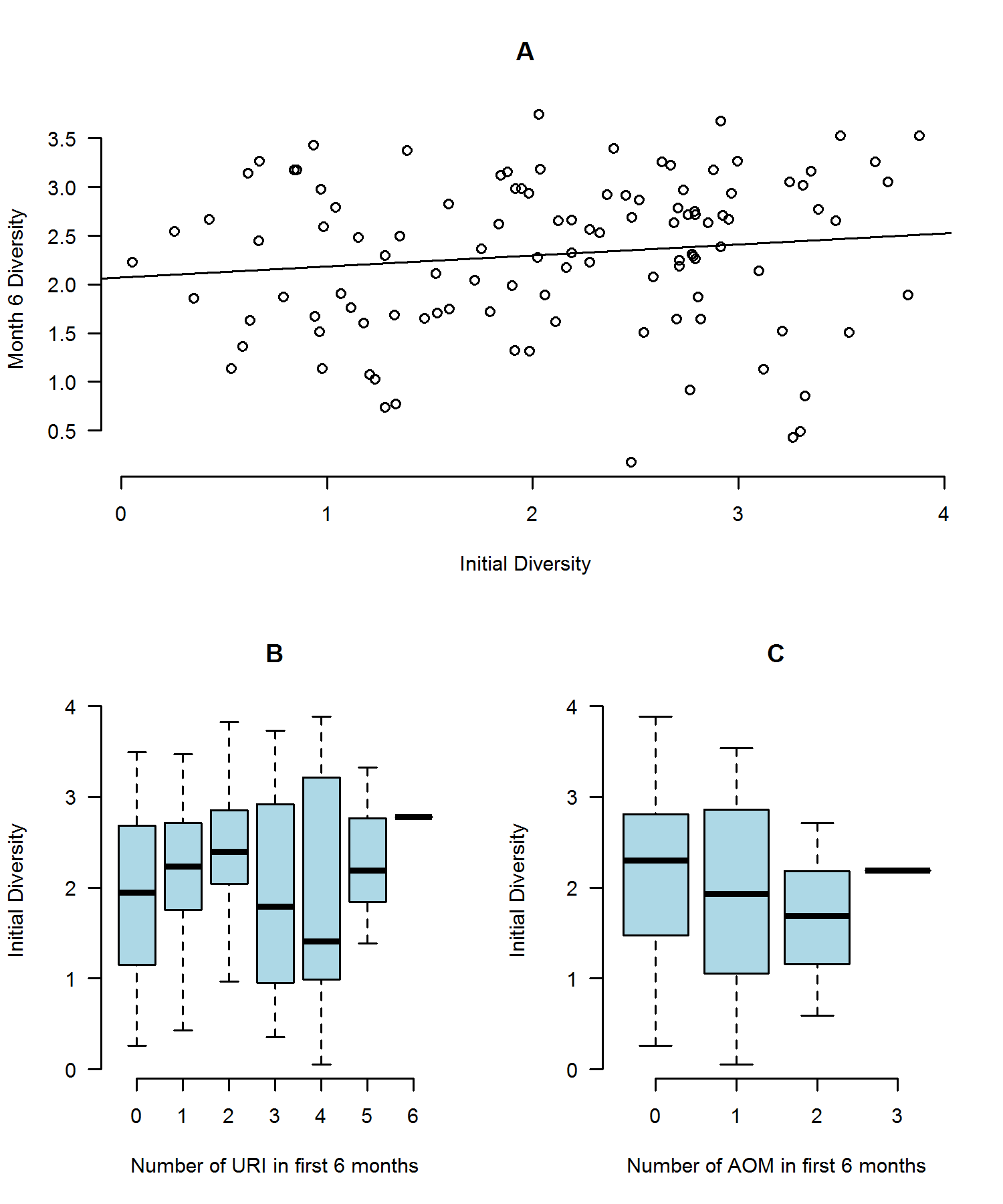

Supplement: S1 Fig — A. Association between baseline diversity and diversity at later age (month 6), P = 0.02. B. Association between diversity at baseline (month 1) and number of URI in the first 6 months. Higher diversity at month 1 was associated with increasing frequencies of URI within the first 6 months (P = 0.036). C. Association between diversity at baseline (month 1) and number of URI in the first 6 months (P = 0.55). (TIF) [file pone.0180630.s001.tif]

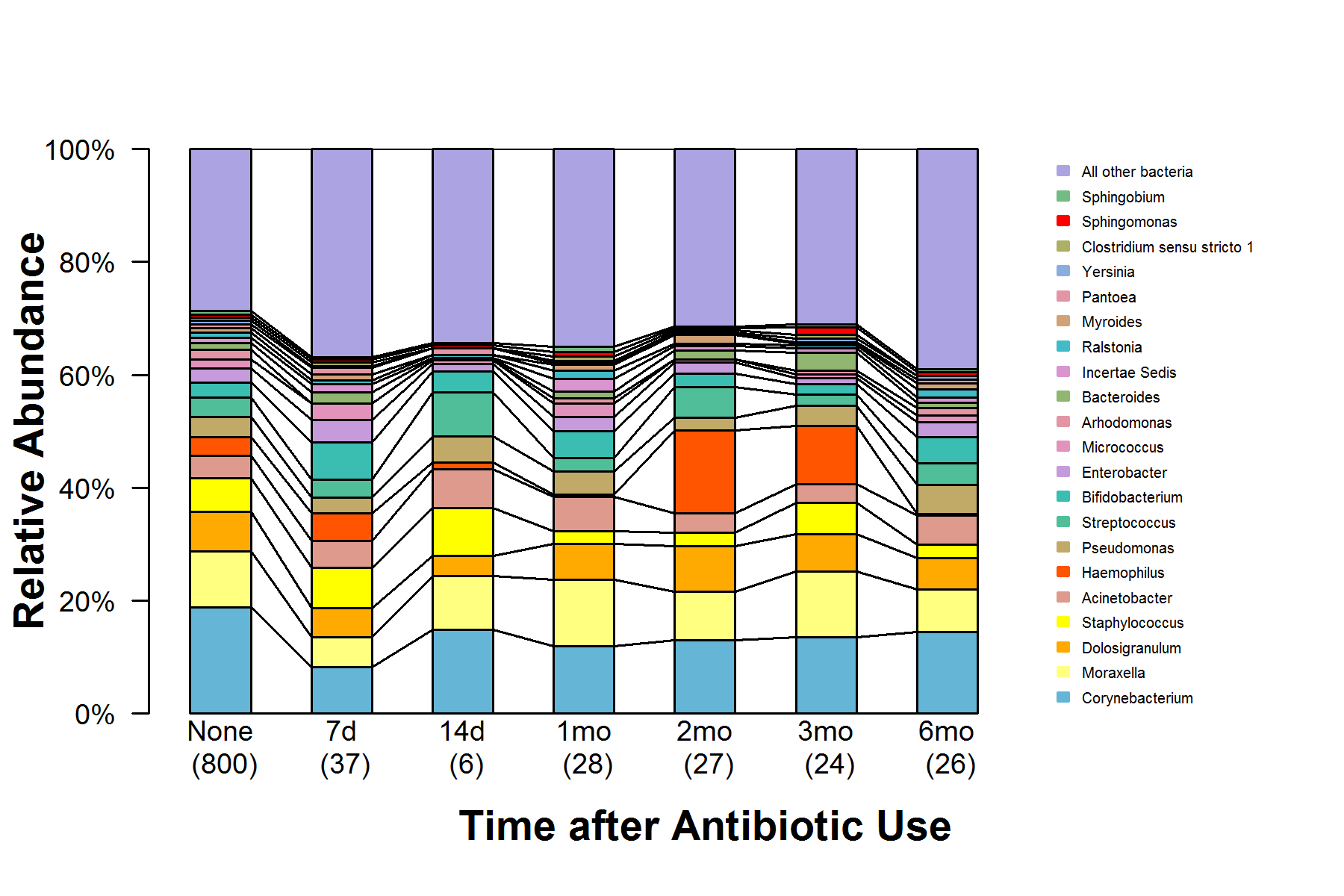

Supplement: S2 Fig — The time point indicates days or months of antibiotic use prior to nasopharyngeal sample collection. Numbers in parentheses are number of samples collected within specific time of antibiotic use. (TIF) [file pone.0180630.s002.tif]
