# Supplementary material for: Nasopharyngeal microbiota in infants and changes during viral upper respiratory tract infection and acute otitis media
Source: PLoS One. 2017 Jul 14;12(7):e0180630. doi: 10.1371/journal.pone.0180630 (PMC5510840; doi:10.1371/journal.pone.0180630)
Supplement: S1 Table — (DOC) [file pone.0180630.s003.doc]

Table S3. Susceptibility to URI and AOM and trends in microbial genera change during in the first 6 months of life^a^

| **Event in the first 6 months** | **Microbial Genus** | **Trend** | **Linear trend P-value** |
| --- | --- | --- | --- |
| Increased number of URI in 3 moths | *Streptococcus* | Increasing^b^ | 0.0112 |
|  |  |  |  |
| Increased number of URI in 6 moths | *Moraxella* | Increasing | 0.0332 |
|  | *Haemophilus* | Increasing | 0.0084 |
|  |  |  |  |
| Increased number of AOM in 6 moths | *Micrococcus* | Decreasing | 0.0257 |
|  |  |  |  |
| a-Data from samples collected from ages 1-6 months; only significant trends are reported | | |  |
| b-Increasing relative abundance of bacteria |  |  |  |
