# Supplementary material for: Nasopharyngeal microbiota in infants and changes during viral upper respiratory tract infection and acute otitis media
Source: PLoS One. 2017 Jul 14;12(7):e0180630. doi: 10.1371/journal.pone.0180630 (PMC5510840; doi:10.1371/journal.pone.0180630)
Supplement: S2 Table — (DOC) [file pone.0180630.s004.doc]

S4 Table. Microbiome composition in URI samples that were complicated by AOM vs URI that resolved^a^

| **Genus** | **Total** | **URI samples with** | **URI samples with** | **P-value** |
| --- | --- | --- | --- | --- |
|  |  | **resolution** | **AOM complication** | **(age-adjusted)** |
|  | (N=184) | (N=167) | (N=17) |  |
| Corynebacterium | 15.9% | 16.1% | 14.0% | 0.1597 |
| Moraxella | 13.2% | 13.4% | 11.8% | 0.7782 |
| Dolosigranulum | 7.8% | 8.0% | 5.7% | 0.3366 |
| Staphylococcus | 5.5% | 5.6% | 4.3% | 0.7091 |
| Haemophilus | 5.0% | 4.5% | 10.7% | 0.2448 |
| Streptococcus | 3.8% | 3.6% | 6.3% | 0.0548 |
| Acinetobacter | 3.2% | 3.1% | 4.0% | 0.8763 |
| Bifidobacterium | 3.0% | 3.1% | 1.3% | 0.1233 |
| Pseudomonas | 2.9% | 3.0% | 2.0% | 0.2668 |
| Enterobacter | 2.2% | 2.2% | 2.3% | 0.3914 |
| Micrococcus | 1.8% | 1.8% | 1.8% | 0.6236 |
| Arhodomonas | 1.2% | 1.3% | 0.6% | 0.3771 |
| Incertae Sedis | 1.0% | 1.0% | 0.4% | 0.3855 |
| Bacteroides | 0.9% | 1.0% | 0.3% | 0.1846 |
| Ralstonia | 0.7% | 0.7% | 0.6% | 0.6089 |
| Sphingomonas | 0.6% | 0.6% | 0.7% | 0.3920 |
| Myroides | 0.5% | 0.6% | 0.2% | 0.5124 |
| Yersinia | 0.5% | 0.5% | 0.4% | 0.5738 |
| Sphingobium | 0.4% | 0.4% | 0.3% | 0.7928 |
| Clostridium sensu stricto 1 | 0.4% | 0.4% | 0.1% | 0.7512 |
| Pantoea | 0.4% | 0.4% | 0.1% | 0.4766 |

* Significant results (at the 0.05 level) after adjustment for multiple testing
