# Supplementary material for: Nasopharyngeal microbiota in infants and changes during viral upper respiratory tract infection and acute otitis media
Source: PLoS One. 2017 Jul 14;12(7):e0180630. doi: 10.1371/journal.pone.0180630 (PMC5510840; doi:10.1371/journal.pone.0180630)
Supplement: S3 Table — (DOC) [file pone.0180630.s005.doc]

S5 Table. Changes in microbiome between paired samples: URI - resolved vs URI to AOM

| **Genus** | **Total** | **Group 1 samples^a^** | **Group 2 samples^b^** | **Odds Ratio** | **Group 3 samples^a^** | **Group 4 samples^d^** | **Odds Ratio** | **P-value^e^** |
| --- | --- | --- | --- | --- | --- | --- | --- | --- |
|  | (N=50) | (N=14) | (N=14) | **Groups 2 vs 1** | (N=11) | (N=11) | **Groups 4 vs 3** |  |
| Corynebacterium | 16.9% | 14.7% | 18.7% | 1.21 | 12.6% | 21.8% | 2.42 | 0.3979 |
| Moraxella | 10.3% | 6.5% | 5.3% | 0.43 | 15.1% | 16.5% | 0.60 | 0.9284 |
| Staphylococcus | 9.0% | 10.4% | 16.5% | 2.50 | 5.7% | 1.0% | 0.09 | **0.0070** |
| Haemophilus | 8.6% | 6.9% | 5.7% | 1.05 | 8.9% | 14.4% | 2.65 | 0.3293 |
| Dolosigranulum | 5.0% | 4.5% | 5.6% | 0.69 | 3.9% | 5.8% | 1.93 | 0.4622 |
| Streptococcus | 4.7% | 2.5% | 3.0% | 0.95 | 7.5% | 6.9% | 0.53 | 0.4115 |
| Acinetobacter | 3.7% | 5.9% | 2.9% | 0.52 | 3.6% | 2.0% | 0.63 | 0.8229 |
| Micrococcus | 3.0% | 3.6% | 2.4% | 1.75 | 1.9% | 3.9% | 2.04 | 0.7703 |
| Pseudomonas | 2.9% | 3.5% | 2.7% | 0.89 | 2.4% | 3.0% | 1.19 | 0.8415 |
| Bifidobacterium | 2.0% | 2.8% | 1.6% | 1.85 | 1.8% | 1.8% | 0.57 | 0.5123 |
| Enterobacter | 1.6% | 1.2% | 1.4% | 0.92 | 3.4% | 0.6% | 0.78 | 0.6966 |
| Arhodomonas | 1.4% | 1.0% | 0.9% | 0.39 | 0.1% | 3.9% | 87.18 | 0.4655 |
| Myroides | 0.9% | 2.6% | 0.5% | 0.41 | 0.2% | 0.1% | 0.08 | 0.9663 |
| Ralstonia | 0.8% | 1.0% | 1.2% | 0.82 | 0.6% | 0.2% | 0.98 | 0.9909 |
| Yersinia | 0.5% | 0.4% | 1.2% | 1.01 | 0.2% | 0.1% | 0.49 | 0.8456 |
| Bacteroides | 0.5% | 0.4% | 0.2% | 0.59 | 0.5% | 0.7% | 1.71 | 0.7485 |
| Sphingomonas | 0.4% | 0.4% | 0.6% | 1.21 | 0.3% | 0.2% | 0.97 | 0.9026 |
| Incertae Sedis | 0.3% | 0.1% | 0.4% | 3.03 | 0.5% | 0.5% | 0.75 | 0.7548 |
| Clostridium sensu stricto 1 | 0.3% | 0.6% | 0.2% | 0.49 | 0.1% | 0.4% | 3.02 | 0.6039 |
| Sphingobium | 0.3% | 0.0% | 0.8% | 22.37 | 0.3% | 0.1% | 0.43 | **0.0370** |
| Pantoea | 0.1% | 0.1% | 0.4% | 2.86 | 0.0% | 0.1% | 1.64 | 0.5939 |

a-URI samples collected within 7 days of URI that resulted in no AOM

b-URI follow up samples, collected < 7 days later, paired to Group 1 samples

c-URI samples from subjects with AOM within 7 days of this URI episode

d-AOM samples from AOM occurring within 7 days of URI, paired with Group 3 samples

e-Compare the Odd Ratios between Groups 2&1 Vs between Groups 4&3
