# Supplementary material for: Nasopharyngeal microbiota in infants and changes during viral upper respiratory tract infection and acute otitis media
Source: PLoS One. 2017 Jul 14;12(7):e0180630. doi: 10.1371/journal.pone.0180630 (PMC5510840; doi:10.1371/journal.pone.0180630)
Supplement: S1 File — (DOC) [file pone.0180630.s006.doc]

**SUPPLEMENTAL DETAILED METHODS**

**Study Design, Subjects, and Specimens:**

The subjects were part of a prospective, longitudinal study (2008-2014) of infants in the first year of life to evaluate the prevalence and risks for URI and AOM development [14]. Nasopharyngeal (NP) specimens analyzed in this study included available specimens (average 7 / per subject) from approximately an equal number of subjects with and without AOM. Specimen selection occurred before the final follow-up outcome of some study subjects. The study was approved by the University of Texas Medical Branch (UTMB) Institutional Review Board, conformed to the human experimentation guidelines of the United States Department of Health and Human Services.

Healthy infants were enrolled from near birth (< 1 month) and completed the study after the first AOM episode was diagnosed, or at age 12 months without AOM. All subjects were followed to at least 6 months. Data were collected on family history, environmental and risk factors upon enrollment and were updated on every contact. Study personnel made home visits to collect NP swabs at 1, 2, 3, 4, 5, and 6 months, and at 9 months if the subject remained in the study. Parents were instructed to report to the study team when the subject developed cold (URI) symptoms: nasal congestion, rhinorrhea, cough, and/or sore throat, with or without constitutional symptoms such as fever, decreased appetite and restless sleep; time and travel were compensated. Symptomatic subjects were evaluated as soon as possible after URI onset and followed closely for AOM complication. During the URI visit, otoscopic examination and tympanometry were performed and NP specimens were collected; a follow-up visit was scheduled 3-5 days and the parents were encouraged to bring the subject in for examination any time they suspected the infant might have an ear infection. AOM diagnosis was made by trained and experienced otoscopists, based on presentations of acute symptoms (fever, irritability, otalgia), signs of tympanic membrane inflammation (intense redness, bulging, opaque tympanic membrane) and presence of middle ear fluid documented by pneumatic otoscopy and/or tympanometry. For cases diagnosed by other medical professionals, the medical record was reviewed for the following otologic findings: color, opacification, bulging and mobility of the tympanic membranes and presence of middle ear fluid. The study personnel called the parents twice monthly to identify any URI or AOM missed since the last contact, and a comprehensive chart review was performed at the end of the follow-up to capture URI or AOM diagnosed by other providers.

**Specimens, Specimen Processing and Bacterial and viral studies:**

NP swab specimens were collected by trained personnel during the monthly visit by introducing a flocked swab (FLOQSwabs, COPAN, Murrieta, CA) into the nose until resistance was met. The swab was rotated gently 180^0^ and then placed in a 1 ml tube of ESwab transport medium (COPAN Diagnostics, Murrieta, CA) and transported to the lab on ice within 1 hour of collection. An aliquot was sent for bacterial culture; the remaining aliquots were kept frozen at -80°C until testing for viruses and microbiota. During URI or AOM, additional nasopharyngeal secretion (NPS) samples were also collected. Molecular virologic studies were performed by high-throughput quantitative PCR assay to detect 13 respiratory viruses including: adenovirus, bocavirus, coronaviruses 229E, NL63, and OC43, enterovirus, human metapneumovirus, influenza A and B, parainfluenza viruses 1 and 3, respiratory syncytial virus (RSV); and rhinovirus [26].

**DNA Extraction/ Amplification and Sequencing:**

From the NP samples, microbial DNA was extracted using the PowerMicrobiome DNA/RNA Isolation kit (MoBio) in the STARlet platform (Hamilton Robotics). From the extracted DNA, 16S rDNA V4 region amplicons (single index) were produced by PCR and sequenced on the MiSeq platform (Illumina) using the 2x250bp protocol yielding pair-end reads that overlap by ~247 bps. Following sequencing, raw BCL files were retrieved from the MiSeq platform and called into FASTQs by Casava v1.8.3 (Illumina). The read pairs were demultiplexed based on unique molecular barcodes allowing for up to 1 substitution mismatch and reconstituted into two FASTQ files for each. The resulting 250 base long paired end reads were merged together based on the overlapping region. These works were performed at the Alkek Center for Metagenomics and Microbiome Research, Baylor College of Medicine (Joseph Petrosino, Director).

**Sequence Analysis:**

To identify the presence of known bacteria and archaea, subsequences were analyzed using CLC Genomics Workbench 8.0.1 Microbial Genomics Module (http://www.clcbio.com). Filtration was done to assure that only high quality sequences would be included in the downstream analysis. During the filtration reads containing nucleotides below the quality threshold of 0.05 (using modified Richard Mott algorithm), reads with two or more unknown nucleotides and sequencing adapters trimmed. All reads were trimmed to 240 bases for operation taxonomic unit (OTU) classification. Reference based OTU picking was performed using the SILVA SSU v119 97% database. Sequences present in more than one copy but not clustered to the database were then placed into de novo OTUs (97% similarity) and aligned against the reference database with 80% similarity threshold. Chimeras were removed from the results if their absolute crossover cost was 3 using a k-mer size of 6.

**Statistics:**

All analyses were done at the genus level. Shannon diversity index was calculated using the entropy function in the entropy library in R statistical package (cran.r-project.org). To account for the within-subject variability component, a mixed model with a random intercept for subject was used for most results (using the lme4 library in R). For instance, bacterial culture status (by sample) was characterized with a logistic regression mixed model. Finally, abundance was calculated using the mean relative abundance across samples.

In comparing relative abundances among samples, we used a linear mixed model on the approximate log relative abundance, i.e., the term given as log [(number of reads for the specific genus+1) / (total number of reads in the sample)]. This term was used to avoid having log (0) as a response value. We compared this to a binomial approach weighted so that each sample contributed equally to the model; while the binomial model was conceptually sound, the fitting of the model was occasionally unstable, leading to unreliable results. It was found that the using approximate log of relative abundance in the model provided a close approximation (with all correlations between sets of p-values above 0.93) to the binomial approach without the occasional instability of model fitting.

Additionally, in both models of diversity and abundance, the log total number of sequence reads per sample was included as a covariate. As a covariate in models of diversity, the number of reads is typically used as a covariate to account for potential biases related to how the entropy approximation is calculated. The importance of total number of reads as a covariate in comparing abundance was a surprise; however, the effect of the number of reads was highly significant (most were less than 10^-8^) and thus not ignorable. A potential explanation of this effect may have to do with small but nonignorable differential effects across taxonomic units in the amplification process. At the same time, the inclusion this covariate did not point to a bias in the marginal inference as most comparisons became more significant. Significance was declared with P < 0.05. All calculations were done in R (version 3.2.2) and associated libraries (lme4, lmerTest, entropy).
