# Supplementary material for: Nasopharyngeal microbiota in infants and changes during viral upper respiratory tract infection and acute otitis media
Source: PLoS One. 2017 Jul 14;12(7):e0180630. doi: 10.1371/journal.pone.0180630 (PMC5510840; doi:10.1371/journal.pone.0180630)
Supplement: S4 File — Table A. Microbiota in healthy samples compared to URI samples. Table B. Microbiota in healthy samples compared to AOM samples. Table C. Microbiota in URI samples compared to AOM samples. (DOC) [file pone.0180630.s009.doc]

Table A. Microbiota in healthy samples compared to URI samples

| **Genus** | **Total** | **Healthy samples** | **URI samples** | **P-value** |
| --- | --- | --- | --- | --- |
|  | **N=903** | **N=685** | **N=218** | **(age-adjusted)** |
| Corynebacterium | 18.0% | 18.3% | 17.2% | 0.7967 |
| Moraxella | 9.5% | 8.4% | 12.8% | **0.0055** |
| Dolosigranulum | 7.0% | 6.8% | 7.5% | 0.3161 |
| Staphylococcus | 5.9% | 6.2% | 5.0% | 0.1612 |
| Acinetobacter | 4.2% | 4.3% | 3.7% | **0.0347** |
| Pseudomonas | 3.5% | 3.7% | 2.9% | **0.0245** |
| Streptococcus | 3.3% | 3.1% | 3.8% | 0.1283 |
| Haemophilus | 3.1% | 2.7% | 4.6% | 0.2787 |
| Bifidobacterium | 2.9% | 2.8% | 3.3% | 0.2163 |
| Enterobacter | 2.4% | 2.5% | 2.3% | 0.6744 |
| Micrococcus | 1.6% | 1.6% | 1.7% | 0.1058 |
| Arhodomonas | 1.5% | 1.6% | 1.0% | 0.2728 |
| Bacteroides | 1.3% | 1.4% | 0.9% | 0.1756 |
| Incertae Sedis | 1.0% | 1.0% | 0.9% | 0.0840 |
| Ralstonia | 0.9% | 1.0% | 0.8% | 0.8180 |
| Myroides | 0.9% | 0.9% | 0.7% | **0.0177** |
| Pantoea | 0.6% | 0.7% | 0.4% | 0.2425 |
| Yersinia | 0.6% | 0.7% | 0.5% | **0.0066** |
| Clostridium sensu stricto 1 | 0.6% | 0.6% | 0.4% | 0.2307 |
| Sphingomonas | 0.6% | 0.6% | 0.6% | 0.0727 |
| Sphingobium | 0.6% | 0.6% | 0.6% | 0.1513 |

* Significant results (at the 0.05 level) after adjustment for multiple testing

Table B. Microbiota in healthy samples compared to AOM samples

| **Genus** | **Total** | **Healthy samples** | **AOM samples** | **P-value** |
| --- | --- | --- | --- | --- |
|  | **N=730** | **N=685** | **N=45** | **(Age-adjusted)** |
| Corynebacterium | 17.9% | 18.3% | 12.5% | 0.5370 |
| Moraxella | 8.8% | 8.4% | 14.2% | **0.0028*** |
| Dolosigranulum | 6.6% | 6.8% | 4.6% | 0.7916 |
| Staphylococcus | 6.0% | 6.2% | 2.1% | 0.1723 |
| Acinetobacter | 4.2% | 4.3% | 3.2% | 0.7336 |
| Pseudomonas | 3.7% | 3.7% | 2.9% | 0.1188 |
| Haemophilus | 3.4% | 2.7% | 14.4% | **0.0002*** |
| Streptococcus | 3.4% | 3.1% | 6.7% | **0.0117** |
| Bifidobacterium | 2.8% | 2.8% | 3.9% | 0.7320 |
| Enterobacter | 2.5% | 2.5% | 3.0% | 0.3077 |
| Arhodomonas | 1.7% | 1.6% | 2.7% | 0.1403 |
| Micrococcus | 1.6% | 1.6% | 2.0% | 0.4379 |
| Bacteroides | 1.4% | 1.4% | 1.1% | 0.2589 |
| Incertae Sedis | 1.0% | 1.0% | 1.0% | 0.2754 |
| Ralstonia | 0.9% | 1.0% | 0.6% | 0.2815 |
| Myroides | 0.9% | 0.9% | 0.4% | 0.0920 |
| Pantoea | 0.7% | 0.7% | 0.7% | 0.7518 |
| Yersinia | 0.7% | 0.7% | 0.7% | **0.0199** |
| Clostridium sensu stricto 1 | 0.6% | 0.6% | 0.5% | 0.9567 |
| Sphingomonas | 0.6% | 0.6% | 0.2% | 0.2306 |
| Sphingobium | 0.6% | 0.6% | 0.5% | 0.9613 |

* Significant results (at the 0.05 level) after adjustment for multiple testing

Table C. Microbiota in URI samples compared to AOM samples

| **Genus** | **Total** | **URI samples** | **AOM samples** | **P-value** |
| --- | --- | --- | --- | --- |
|  | **N=263** | **N=218** | **N=45** | **(Age-adjusted)** |
| Corynebacterium | 16.4% | 17.2% | 12.5% | 0.2481 |
| Moraxella | 13.1% | 12.8% | 14.2% | 0.1145 |
| Dolosigranulum | 7.0% | 7.5% | 4.6% | 0.9819 |
| Haemophilus | 6.3% | 4.6% | 14.4% | **0.0033** |
| Staphylococcus | 4.5% | 5.0% | 2.1% | 0.5607 |
| Streptococcus | 4.3% | 3.8% | 6.7% | 0.0652 |
| Acinetobacter | 3.7% | 3.7% | 3.2% | 0.6357 |
| Bifidobacterium | 3.4% | 3.3% | 3.9% | 0.7950 |
| Pseudomonas | 2.9% | 2.9% | 2.9% | 0.3708 |
| Enterobacter | 2.4% | 2.3% | 3.0% | 0.6933 |
| Micrococcus | 1.8% | 1.7% | 2.0% | 0.6203 |
| Arhodomonas | 1.3% | 1.0% | 2.7% | 0.0980 |
| Incertae Sedis | 1.0% | 0.9% | 1.0% | 0.5856 |
| Bacteroides | 0.9% | 0.9% | 1.1% | 0.9810 |
| Ralstonia | 0.8% | 0.8% | 0.6% | 0.0369 |
| Myroides | 0.6% | 0.7% | 0.4% | 0.5666 |
| Sphingobium | 0.6% | 0.6% | 0.5% | 0.9639 |
| Sphingomonas | 0.5% | 0.6% | 0.2% | 0.5960 |
| Yersinia | 0.5% | 0.5% | 0.7% | **0.0451** |
| Clostridium sensu stricto 1 | 0.4% | 0.4% | 0.5% | 0.3345 |
| Pantoea | 0.4% | 0.4% | 0.7% | 0.3614 |

* Significant results (at the 0.05 level) after adjustment for multiple testing
