# Supplementary material for: Nasopharyngeal microbiota in infants and changes during viral upper respiratory tract infection and acute otitis media
Source: PLoS One. 2017 Jul 14;12(7):e0180630. doi: 10.1371/journal.pone.0180630 (PMC5510840; doi:10.1371/journal.pone.0180630)
Supplement: S5 File — Table A. Microbiota in virus-positive healthy samples and virus-positive URI samples. Table B. Microbiota in virus-negative healthy samples and virus-positive URI samples. Table C. Effect of asymptomatic virus infection: microbiota in healthy virus-negative vs healthy virus-positive samples. Table D. Microbiota in rhinovirus-negative samples and rhinovirus-positive samples. (DOC) [file pone.0180630.s010.doc]

Table A. Relative abundance of microbiota in virus-positive healthy samples and virus-positive URI samples

| **Genus** | **All virus-positive samples** | **Virus-positive healthy samples** | **Virus-positive URI samples** | **P-value** |
| --- | --- | --- | --- | --- |
|  | **N=372** | **N=202** | **N=170** | **(age-adjusted)** |
| Corynebacterium | 16.3% | 17.5% | 15.0% | 0.2972 |
| Moraxella | 12.0% | 10.5% | 13.8% | **0.0050** |
| Dolosigranulum | 6.3% | 6.4% | 6.2% | 0.6033 |
| Haemophilus | 5.6% | 3.6% | 7.9% | **0.0163** |
| Staphylococcus | 5.2% | 6.2% | 4.0% | 0.6675 |
| Streptococcus | 4.6% | 4.5% | 4.7% | 0.4458 |
| Acinetobacter | 3.9% | 4.4% | 3.2% | 0.1668 |
| Pseudomonas | 3.4% | 3.8% | 3.0% | 0.0524 |
| Bifidobacterium | 3.3% | 2.8% | 3.9% | 0.5183 |
| Enterobacter | 2.5% | 2.5% | 2.4% | 0.6065 |
| Micrococcus | 1.5% | 1.4% | 1.6% | 0.3159 |
| Incertae Sedis | 1.2% | 1.2% | 1.2% | 0.3415 |
| Bacteroides | 1.1% | 1.2% | 1.0% | 0.3417 |
| Arhodomonas | 1.0% | 1.1% | 0.8% | 0.5244 |
| Ralstonia | 0.8% | 0.8% | 0.7% | 0.3482 |
| Myroides | 0.6% | 0.8% | 0.5% | 0.0891 |
| Yersinia | 0.6% | 0.6% | 0.6% | 0.1280 |
| Pantoea | 0.6% | 0.8% | 0.4% | 0.6097 |
| Sphingobium | 0.6% | 0.7% | 0.5% | **0.0412** |
| Sphingomonas | 0.5% | 0.5% | 0.6% | 0.5924 |
| Clostridium sensu stricto 1 | 0.5% | 0.5% | 0.5% | 0.5292 |

* Significant results (at the 0.05 level) after adjustment for multiple testing

Table B. Relative abundance of microbiota in virus-negative healthy samples and virus-positive URI samples

| **Genus** | **Total** | **Virus-negative healthy samples^b^** | **Virus-positive URI**  **samples^a^** | **P-value** |
| --- | --- | --- | --- | --- |
|  | **N=613** | **N=443** | **N=170** | **(age-adjusted)** |
| Corynebacterium | 17.6% | 18.6% | 15.0% | 0.4531 |
| Moraxella | 9.5% | 7.9% | 13.8% | **<0.0001*** |
| Dolosigranulum | 6.9% | 7.2% | 6.2% | 0.4869 |
| Staphylococcus | 5.8% | 6.5% | 4.0% | 0.7680 |
| Acinetobacter | 4.0% | 4.3% | 3.2% | 0.1005 |
| Haemophilus | 4.0% | 2.4% | 7.9% | **0.0137** |
| Pseudomonas | 3.5% | 3.7% | 3.0% | 0.2286 |
| Streptococcus | 3.2% | 2.6% | 4.7% | **0.0031*** |
| Bifidobacterium | 3.1% | 2.8% | 3.9% | 0.7735 |
| Enterobacter | 2.5% | 2.6% | 2.4% | 0.5127 |
| Micrococcus | 1.6% | 1.6% | 1.6% | 0.5638 |
| Bacteroides | 1.4% | 1.5% | 1.0% | 0.3137 |
| Arhodomonas | 1.4% | 1.6% | 0.8% | 0.6269 |
| Incertae Sedis | 1.0% | 0.9% | 1.2% | 0.3106 |
| Ralstonia | 1.0% | 1.0% | 0.7% | 0.9199 |
| Myroides | 0.9% | 1.1% | 0.5% | **0.0072** |
| Clostridium sensu stricto 1 | 0.7% | 0.7% | 0.5% | 0.7229 |
| Yersinia | 0.6% | 0.6% | 0.6% | 0.0655 |
| Sphingomonas | 0.6% | 0.6% | 0.6% | 0.4380 |
| Pantoea | 0.6% | 0.7% | 0.4% | 0.1863 |
| Sphingobium | 0.5% | 0.5% | 0.5% | 0.9401 |
| a- Of 264 healthy samples, virus data were available in 245 (93%); 75 samples were virus-negative | | | |  |
| b- Of 684 URI samples, virus data were available in 645 (94%); 202 samples were virus-positive  * Significant results (at the 0.05 level) after adjustment for multiple testing | | | |  |

Table C. Effect of asymptomatic virus infection: microbiota in healthy virus-negative vs healthy virus-positive samples

| **Genus** | **All healthy samples^a^** | **Virus-negative samples** | **Virus-positive samples** | **P-value** |
| --- | --- | --- | --- | --- |
|  | **N=645** | **N=443** | **N=202** | **(age-adjusted)** |
| Corynebacterium | 18.3% | 18.6% | 17.5% | 0.2220 |
| Moraxella | 8.7% | 7.9% | 10.5% | 0.9116 |
| Dolosigranulum | 6.9% | 7.2% | 6.4% | 0.6342 |
| Staphylococcus | 6.4% | 6.5% | 6.2% | 0.7493 |
| Acinetobacter | 4.3% | 4.3% | 4.4% | 0.7635 |
| Pseudomonas | 3.7% | 3.7% | 3.8% | 0.3358 |
| Streptococcus | 3.2% | 2.6% | 4.5% | **0.0474** |
| Bifidobacterium | 2.8% | 2.8% | 2.8% | 0.8934 |
| Haemophilus | 2.8% | 2.4% | 3.6% | 0.5931 |
| Enterobacter | 2.6% | 2.6% | 2.5% | 0.8218 |
| Micrococcus | 1.5% | 1.6% | 1.4% | 0.5142 |
| Arhodomonas | 1.4% | 1.6% | 1.1% | 0.4700 |
| Bacteroides | 1.4% | 1.5% | 1.2% | 0.0566 |
| Incertae Sedis | 1.0% | 0.9% | 1.2% | 0.8786 |
| Myroides | 1.0% | 1.1% | 0.8% | 0.3065 |
| Ralstonia | 1.0% | 1.0% | 0.8% | 0.1422 |
| Pantoea | 0.7% | 0.7% | 0.8% | 0.4972 |
| Yersinia | 0.6% | 0.6% | 0.6% | 0.7883 |
| Clostridium sensu stricto 1 | 0.6% | 0.7% | 0.5% | 0.7910 |
| Sphingobium | 0.6% | 0.5% | 0.7% | 0.9070 |
| Sphingomonas | 0.6% | 0.6% | 0.5% | **0.0260** |
| a - Of 684 healthy samples, virus data were available in 645 (94%)  * Significant results (at the 0.05 level) after adjustment for multiple testing | | |  |  |

Table D. Relative abundance of microbiota in rhinovirus-negative samples and rhinovirus-positive samples

| **Genus** | **All samples** | **Rhinovirus-negative samples** | **Rhinovirus-positive samples** | **P-value** |
| --- | --- | --- | --- | --- |
|  | **N=890** | **N=697** | **N=193** | **(age-adjusted)** |
| Corynebacterium | 17.8% | 18.5% | 15.1% | 0.3760 |
| Moraxella | 9.9% | 9.3% | 12.1% | 0.8702 |
| Dolosigranulum | 6.9% | 7.2% | 5.9% | 0.8152 |
| Staphylococcus | 5.9% | 6.0% | 5.7% | 0.5959 |
| Acinetobacter | 4.1% | 4.2% | 3.8% | 0.4684 |
| Haemophilus | 3.8% | 2.8% | 7.2% | **0.0217** |
| Pseudomonas | 3.5% | 3.6% | 3.3% | 0.5420 |
| Streptococcus | 3.5% | 3.1% | 4.7% | 0.5378 |
| Bifidobacterium | 3.0% | 2.9% | 3.2% | 0.6121 |
| Enterobacter | 2.5% | 2.6% | 2.2% | 0.2479 |
| Micrococcus | 1.6% | 1.6% | 1.7% | 0.7627 |
| Arhodomonas | 1.3% | 1.3% | 1.3% | 0.0632 |
| Bacteroides | 1.3% | 1.4% | 0.8% | 0.1348 |
| Incertae Sedis | 1.0% | 0.9% | 1.2% | 0.6703 |
| Ralstonia | 0.9% | 0.9% | 0.8% | 0.8546 |
| Myroides | 0.9% | 1.0% | 0.6% | 0.8268 |
| Pantoea | 0.6% | 0.6% | 0.7% | 0.4214 |
| Yersinia | 0.6% | 0.6% | 0.6% | 0.2355 |
| Clostridium sensu stricto 1 | 0.6% | 0.6% | 0.5% | 0.6778 |
| Sphingomonas | 0.6% | 0.6% | 0.6% | 0.5052 |
| Sphingobium | 0.6% | 0.5% | 0.6% | 0.5036 |

* Significant results (at the 0.05 level) after adjustment for multiple testing
